# Gender sensitivity in nursing practice: assessing the impact of childhood experiences of domestic violence and perceptions of sexism among healthcare providers on their gender sensitivity

**DOI:** 10.1186/s12912-024-02056-y

**Published:** 2024-06-11

**Authors:** Sun Jeong Yun, Hye Young Kim

**Affiliations:** College of Nursing, Keimyung University, Daegu, Republic of Korea

**Keywords:** Nurses, Domestic violence, Sexism, Gender sensitivity

## Abstract

**Background:**

Gender sensitivity, which is the capacity to recognize and address issues of gender discrimination and inequality, is initiated with an awareness of gender differences. This is particularly crucial in nursing, where care is tailored to the holistic needs of individuals. Given the sensitive nature of nursing to gender variances, it is essential that the influences of nurses’ own experiences and perceptions on their gender sensitivity are explored. This study is aimed at assessing the effects of childhood experiences of domestic violence and perceptions of sexism among healthcare providers on their gender sensitivity. Additionally, it seeks to provide empirical data to support the enhancement of gender-sensitive practices within nursing environments, thereby fostering a culture of gender equality, and helping to promote the practical application of gender equality within nursing organizations.

**Methods:**

A cross-sectional survey was employed to gather data from 146 nurses aged 24 and above residing in Daegu. The general characteristics of these nurses, their childhood experiences of domestic violence, their perceptions of sexism, and their level of gender sensitivity were measured. The data were then subjected to a series of statistical analyses, including t-tests, one-way analysis of variance, Pearson’s correlation coefficients, and hierarchical regression analysis, to identify the factors influencing gender sensitivity.

**Results:**

It was revealed by the analysis that nurses’ gender sensitivity was not significantly associated with their childhood experiences of domestic violence. However, a negative correlation was found between gender sensitivity and their perceptions of sexism (*r* = -0.46, *p* < 0.001). Additionally, age and perception of sexism were emerged as significant predictors of gender sensitivity, accounting for 42.7% of the variance in the regression model.

**Conclusion:**

This study identifies age and sexism perceptions as key predictors of gender sensitivity among nurses, accounting for 42.7% of the variance. It highlights the importance of recognizing generational cultural differences and implementing flexible practices in nursing organizations. Leaders should enhance cultural awareness and address sexism. Further research is needed on the role of societal and cultural norms in recognizing domestic violence. These findings emphasize the need for targeted interventions to improve gender sensitivity and support high-quality nursing care.

## Background

Gender sensitivity is defined as the capacity to recognize the problem of ‘gender differences’ that are created socially, culturally, and historically. It is also characterized by the attitude of acknowledging and respecting diversity in gender without prejudice [1]. This concept is highlighted as important for understanding and addressing gender discrimination and inequality, as well as for promoting an inclusive and unbiased approach towards various gender identities [2].

Nurses are healthcare professionals whose maintenance and enhancement of patient health are based on their professional expertise, and they play a crucial role in disease prevention and health promotion activities [3]. At this time, interactions with patients of all ages and genders are conducted by nurses, who must be aware of the patients’ gender, cultural, and social backgrounds [4]. Within the rapidly evolving healthcare environment, informatization, and increasing focus on customer-centric care, nurses are often unilaterally perceived as performing ‘women’s labor’ due to strong societal stereotypes [5]. This perception necessitates a new professional reflection on gender roles, gender identity, and gender equality in line with contemporary changes. According to the U.S. Bureau of Labor Statistics in 2023, the percentage of male nurses has been surpassed by 12%, and globally, the proportion of male nurses is steadily increasing [6].

In addition to embracing gender diversity within the nursing profession, sensitive healthcare services are being provided by nurses due to the nature of the health and medical field, which often involves gender-specific needs. The need for gender sensitivity is seen as crucial, as nursing involves holistic care based on a deep understanding of human beings [7, 8]. Consequently, it is recommended in practice that gender education be received by nursing students during their university coursework. However, most of this education, except for sexual violence prevention, focuses on biological sex without adequately considering social gender differences [9].

Gender sensitivity extends beyond just the acquisition of knowledge or skills; it is related to emotions and values that contribute to gender awareness [10]. Gender sensitivity is recognized by the World Health Organization (WHO) as a crucial social determinant of health, and strategic interventions in childhood and adolescence are advocated [11, 12]. An act of harm caused by physical and emotional (including verbal, sexual, and economic) violence between family members is defined as a childhood experience of domestic violence. Aggressiveness and strong traditional gender role stereotypes are often exhibited by individuals with such experiences. They react unfavorably to different gender roles for men and women and have an attitude of strictly dichotomizing them based on biological sex [13]. This attitude can lead to immature responses in medical settings where gender differences must be recognized and can further negatively impact nursing work. Accordingly, an examination of nurses’ childhood experiences of domestic violence is proposed.

Perception of sexism is defined as the ability to recognize emotional discomfort regarding gender dichotomy and assists individuals in feeling the severity of gender discrimination issues, examining gender-sensitive perspectives in relationships with others, and exploring the possibility of change to desirable relationships [14]. The absence of perception of sexism is reinforced by rigid views of different genders and can be a major factor in causing gender role conflict [15]. Respect for all patients and the provision of nursing care without prejudice are facilitated by the perception of sexism [16], and it is emerging as an important issue within the nursing profession, especially as the proportion of male nurses increases. Various areas such as department placement, job evaluation, marriage, pregnancy, or childbirth can be impacted by this [17].

Previous research on gender sensitivity has been conducted across various academic fields. In the legal profession, gender sensitivity has been confirmed as an important rational factor in trials of sexual violence crimes [18], and in the field of social pedagogy, it has been found that parents’ gender role stereotypes are related to influencing adolescent children and affecting their gender sensitivity [19]. In a systematic literature review study, it was found that gender sensitivity training interventions for healthcare providers, including education on gender and sex terminology, lectures and discussions on bias and discrimination, and the use of online platforms for training, resulted in significant improvements in gender-related knowledge and attitudes [20]. Furthermore, where gender sensitivity training for medical students is actively underway in the medical field, relatively high levels of gender sensitivity are exhibited by medical students, as indicated by results from online surveys [21]. This education is necessary as it equips medical students to provide patient-centered care and reduce biases related to gender. However, gender sensitivity in nursing has been scarcely examined. The lack of research is thought to be due to the prioritization of studies on clinical skills, patient management, and the improvement of medical quality [22], while the importance of social awareness, such as gender sensitivity, has not been adequately considered.

The aims of this study are to understand the impact of childhood domestic violence experiences and perceptions of sexism on nurses’ gender sensitivity, and to propose strategies for the real dissemination of gender equality concepts among nurses and nursing students, as well as to provide foundational data for the development of gender sensitivity training programs.

The following hypotheses were proposed:

### Hypothesis 1

Nurses’ gender sensitivity will have a negative correlation with experiences of domestic violence in childhood.

### Hypothesis 2

Nurses’ gender sensitivity will have a negative correlation with perceptions of sexism.

### Hypothesis 3

Experiences of domestic violence in childhood and perceptions of sexism are expected to affect nurses’ gender sensitivity.

## Methods

### Study design

A correlational research design was used in this study to determine the effects of childhood experience of domestic violence and perceptions of sexism on nurses’ gender sensitivity. It was based on the STROBE (STrengthening the Reporting of OBservational studies in Epidemiology) reporting guidelines.

### Study population and sampling procedure

This study was conducted with nurse practitioners currently working in hospitals, general hospitals, and senior general hospitals in Daegu Metropolitan City. The criteria for participant selection in this study are as follows: participants must be at least 24 years old, which is the average age at which individuals in Korea are employed as nurses after completing the formal nursing program and passing the national examination. Additionally, participants must be able to understand and respond to the questionnaire, capable of communication, aware of the study’s purpose, and willing to consent to participate. The number of participants was calculated using the G.Power 3.1.2 program, based on the prior study by Wang, Sarker, Carbonetto, and Stephens [23]. Considering a medium effect size of 0.15, a significance level of 0.05, a power of 0.90, and nine predictor variables in a multiple regression analysis, a total of 134 participants were deemed necessary. To account for a 10% dropout rate, questionnaires were distributed to 149 nurses, and all but three were returned; thus, a total of 146 responses were utilized in the final data analysis.

### Measurements

The structured instrument designed to measure the key variables in this study was sent via email to the developers to obtain their approval for use.

#### General characteristics

Based on previous studies, a total of nine questions investigated characteristics related to gender, age, marital status, education, position, work experience, work department, and experience in gender-sensitive education and methods.

#### Childhood experience of domestic violence

The Pare nt–Child Conflict Scale (PCCTS) by Straus, Hamby, Finkelhor, Moore, and Runyan [24] and a modified version by Choi [25] were used without modifications to measure childhood experiences of domestic violence. Choi [25] excluded the neglect portion of the PCCTS as well as items related to emotional and physical abuse that were not appropriate for Korean culture. This scale consisted of 14 questions with five questions on emotional abuse and nine on physical abuse. Each question was scored on a 5-point Likert scale, with higher scores indicating higher levels of abuse in that area. In Choi’s [25] study, the reliability of the instrument was as follows: Cronbach’s α = 0.78 for emotional abuse and Cronbach’s α = 0.79 for physical abuse. In this study, it was Cronbach’s α = 0.77 for emotional abuse and Cronbach’s α = 0.81 for physical abuse.

#### Perception of sexism

A translation of the Sexism Scale developed by Swim, Aikin, Hall, and Hunter [26] was used. The scale comprises items that cover traditional gender roles, differential treatment of men and women, and acceptance of stereotypes about women’s lesser abilities, as well as items that measure denial of the persistence of discrimination against women, hostility to women’s needs, and lack of support for policies aimed at helping women. A total of 13 questions were scored on a 7-point Likert scale, with A higher score in perception of sexism indicates that an individual or group holds a discriminatory perspective based on gender, which means they harbor specific prejudices or stereotypes related to gender. The reliability of the research instrument was Cronbach’s α = 0.83, and in this study, it was Cronbach’s α = 0.79.

#### Gender sensitivity

To measure gender sensitivity, an instrument developed by Lee [1] was used. The tool consists of 31 questions classified under four subsections: 7 questions on openness to gender identity, 8 on self-reflection, 7 on nonviolence, and 9 on gender roles. Of these, 19 negative questions were reverse scaled. Each question was scored on a 5-point Likert scale, with higher scores indicating greater gender sensitivity. The reliability of the instrument for each factor in the studies on nursing students by Lee [1] and Ju and Lee [27] was Cronbach’s α = 0.84. In this study, it was Cronbach’s α = 0.82.

### Data collection

Data were collected from November 1, 2022, to January 10, 2023. The participants, nurse practitioners currently working in hospitals, general hospitals, and senior general hospitals in Daegu Metropolitan City, who understood the need and purpose of this study and met the eligibility criteria, were given access to the online survey through a quick response code and a uniform resource locator–shortening service. The program was designed such that the online survey could only be accessed by those who consented to the collection of personal information. The questionnaire, requiring approximately 15 min to complete, was answered, and a digital coffee coupon was provided as a reward for completing the study.

### Ethical considerations

To protect the study participants, this study was approved by the institutional review board (IRB) of Keimyung University after deliberation on the purpose, methods, human subject’s protection guarantees, and questionnaires prior to conducting the research (IRB no.: 40525-202204-HR-017-03).

### Data analysis

The collected data were analyzed using IBM SPSS ver. 22.0 (IBM Corp., Armonk, NY, USA), and the detailed methodology is as follows.

1. The general characteristics of the subjects were presented as real numbers and percentages or mean and standard deviation.
2. The degree of Childhood experience of domestic Violence, perception of sexism, and gender sensitivity of the subjects were presented as mean and standard deviation.
3. The differences in gender sensitivity according to the general characteristics of the subjects were analyzed via independent *t*-test and one-way analysis of variance, and post hoc tests were performed using the Scheffé test.
4. Pearson correlation was used to determine the relationship between Childhood experience of domestic Violence, perception of sexism, and gender sensitivity. Furthermore, according to Cohen’s criteria for correlation effect size, *r* = 0.10 is interpreted as a small effect, *r* = 0.30 as a medium effect, and *r* = 0.50 or higher as a large effect.
5. To identify the factors affecting the participants’ gender sensitivity and to validate the regression model, the first step involved the examination of the scatter plot of the residuals to confirm the linear relationship and homoscedasticity between the dependent and independent variables. It was confirmed that the assumptions of linearity and homoscedasticity of the model were met. Secondly, the independence of the residuals was assessed using the Durbin-Watson statistic; an index value of 1.682 confirmed that there was no autocorrelation among the error terms. Thirdly, the normality of all variables was examined by conducting the Shapiro-Wilk test, confirming that all variables followed a normal distribution as their significance levels were higher than 0.05. Lastly, the correlation between independent variables was evaluated by analyzing the issue of multicollinearity. The results of the Variance Inflation Factor (VIF) indicated that all values were below 10, demonstrating the absence of multicollinearity. Furthermore, the regression model was found to meet the statistical assumptions, allowing the implementation of hierarchical regression analysis.

## Results

### General participant characteristics

Of the participants, 67.1% (98) were female and 32.9% (48) were male; the average age was 35.51 ± 0.75 years; 66.4% (97) had a bachelor’s degree, 22.6% (33) had a master’s degree, and 11.0% (16) had a doctorate; 61.6% (90) were general registered nurses, 49.3% (72) worked in the general ward, 21.2% (31) in the nursing-care integrated service ward, and 21.2% (31) in special departments; and 86.3% (126) responded “Yes” to gender sensitivity training experience (Table 1).

**Table 1:** Differences in Gender sensitivity according to the characteristics of the subjects (*N* = 146)

Variables	Categories	n (%)	Categories (mean ± SD)	Gender sensitivity				
				(mean ± SD)		t or F	*p*	Scheffé
Gender	Female	98(67.1)		2.85 ± 0.15	1.03		0.312	
	Male	48(32.9)		3.09 ± 0.14				
Age (year)	≤ 29^a^	71 (48.6)	35.51 ± 0.75	3.27 ± 0.14		3.84	0.003	a > d
	30-39^b^	32 (21.9)		3.10 ± 0.17				
	40-49^c^	24 (16.4)		3.11 ± 0.14				
	≥ 50^d^	19 (13.0)		3.08 ± 0.17				
Education Degree	Bachelor’s	97(66.4)		3.12 ± 0.14		1.15	0.236	
	Master’s	33(22.6)		3.08 ± 0.17				
	Doctor’s	16(11.0)		3.05 ± 0.12				
Position	Staff Nurse	90(61.6)		3.09 ± 0.15		0.63	0.592	
	Charge Nurse	28(19.2)		3.12 ± 0.16				
	Head Nurse	20(13.7)		3.13 ± 0.14				
	Chief nursing officer	8(5.5)		3.12 ± 0.17				
Unit	General unit	72(49.3)		3.11 ± 0.15		0.31	0.819	
	Comprehensive nursing care unit	31(21.2)		3.08 ± 0.16				
	Special unit	31(21.2)		3.09 ± 0.14				
	Outpatient or other	12(8.2)		3.09 ± 0.16				
Gender sensitivity Education Experience	Yes	126(86.3)		3.27 ± 0.15		2.97	0.011	
	No	20(13.7)		2.90 ± 0.17				

### Participants’ childhood experience of domestic violence, perception of sexism, and gender sensitivity

The average score for Childhood experience of domestic Violence was 2.01 of 5, with a subfactor score of 2.87 for emotional abuse and 1.23 for physical abuse; the average score for the perception of sexism was 3.61 of 5; and the average score for gender sensitivity was 3.17 of 5. The mean ratings of the subscales were 3.24 for openness to gender identity, 3.41 for self-reflection, 3.20 for nonviolence, and 3.22 for openness to gender roles (Table 2).

**Table 2:** Childhood experience of domestic violence, Perception of Sexism, and Gender sensitivity (*N* = 146)

Variables	Min	Max	M ± SD
**Childhood experience of domestic violence**	1.00	3.12	2.01 ± 0.78
Emotional Violence	1.00	5.00	2.87 ± 2.11
Physical Violence	1.00	3.24	1.23 ± 0.32
**perceptions of sexism**	1.00	5.00	3.61 ± 0.30
**Gender sensitivity**	1.21	5.00	3.17 ± 1.21
Open Mindedness to Gender Identity	1.00	5.00	3.24 ± 1.01
Self-Reflection	1.41	5.00	3.41 ± 0.94
Nonviolence	1.00	4.67	3.20 ± 0.88
Open Mindedness to Gender Roles	1.21	5.00	3.22 ± 0.18

‡M ± SD = mean ± standard deviation

### Differences in gender sensitivity based on general participant characteristics

The difference in gender sensitivity according to the general characteristics of the participants was 3.27 for those aged < 30 years and 3.08 for those aged > 50 years, with the former category being more gender sensitive (F = 3.84, *p =* 0.003) and those with gender sensitivity training experience being more gender sensitive (t = 2.97, *p =* 0.011) than those without (Table 1).

### Relationship between the participants’ childhood experience of domestic violence, perception of sexism, and gender sensitivity

Participants’ gender sensitivity showed no statistically significant association with the extent of childhood domestic violence experiences, but there was a statistically significant negative correlation with perceptions of sexism (*r* = − 0.46, *p* < 0.001), corresponding to a medium effect size according to Cohen’s criteria (Table 3).

**Table 3:** Relationships among Childhood experience of domestic violence, Perception of Sexism, and Gender sensitivity (*N* = 146)

Variable	*r* (*p*)		
	CEDV	Perceptions of sexism	Gender Sensitivity
CEDV	1		
perceptions of sexism	0.88 (0.229)	1	
Gender sensitivity	− 0.39 (0.638)	− 0.46 (< 0.001)	1

†CEDV = Childhood experience of domestic violence

### Factors affecting participants’ gender sensitivity

Hierarchical multiple regression analysis was conducted to identify the factors influencing the gender sensitivity of the participants, and the results are shown in Table 4.

**Table 4:** Factors affecting Gender sensitivity (*N* = 146)

Variable	Model 1				Model 2			
	B	β	t	*p*	B	β	t	*p*
**Age (year)**								
30-40^b^	0.19	0.14	1.71	< 0.001	0.19	0.13	1.67	< 0.001
40-50^c^	1.01	0.36	1.11	0.068	1.08	0.24	1.47	0.063
≥ 50^d^	1.21	0.08	0.74	0.460	1.11	0.09	0.94	0.347
**Gender Sensitivity Education Experience**								
No	0.11	0.36	1.24	0.217	0.11	0.34	0.83	0.409
**CEDV**					-0.09	− 0.13	-0.15	0.217
**perceptions of sexism**					-0.23	− 0.48	-3.20	0.002
R^2^	0.281				0.347			
Adjusted R^2^	0.178				0.427			
F (𝑝)	16.10 (< 0.001)				31.70 (< 0.001)			

†CEDV = Childhood experience of domestic violence

‡Reference: Age (year), < 30 = 0, 30–40 = 1, 40–50 = 2, > 50 = 3; Gender Sensitivity Education Experience, Yes = 0, No = 1

Model 1 is an analytical model in which the variables of age and gender sensitivity training experience were introduced simultaneously, as these were common characteristics that were significantly associated with participants’ gender sensitivity. Age was found to influence gender sensitivity (β = 0.14, *p* < 0.001). The regression was significant (F = 16.10, *p* < 0.001), with an explanatory power of 17.8%. For Model 2, the regression model was significant (F = 31.70, *p* < 0.001), with 42.7% explanatory power when the main variables of Childhood experience of domestic Violence and the perception of sexism were entered. Thus, there was a significant increase of 24.9% compared with Model 1. Age (β = 0.13, *p* < 0.001) and the perception of sexism (β = −0.48, *p =* 0.002) had a significant effect on gender sensitivity.

## Discussion

This study was aimed at identifying nurses’ childhood experience of domestic violence and perceptions of sexism and understanding the factors affecting gender sensitivity. By doing so, it was sought to provide foundational data for the development of gender sensitivity training programs for nurses and contribute to the body of research on the topic.

The degree of childhood domestic violence experienced by the subjects of this study was scored at an average of 2.01 out of 5, with emotional abuse rated at 2.87 and physical abuse at 1.23. In the study by Lee and Kim [28], the degree of childhood domestic violence experienced was rated at 1.31 out of 5, with emotional abuse at 1.75 and physical abuse at 1.19. This is different from the study by Lee and Kim [28], which targeted college students under the age of 25, as the subjects in this study were ranged in age from their 20s to over 50s. When the age range is wide, a high likelihood exists that a variety of experiences have been accumulated over a long period of time, and it is thought that older participants are more likely to respond honestly about their experiences.

The average score for the perception of sexism by nurses was measured at 3.61 out of a maximum of 7 points, which is 0.88 points higher than the score found in the study by Jeong [17], which involved only male nurses. This result can be interpreted as an indication that the sensitivity to discriminatory cultures or policies is higher in this study due to the high proportion of female nurses. In the nursing field, where women constitute the majority, the scores on the perception of sexism may have been influenced by sensitivity to gender-based biases and stereotypes.

Gender sensitivity among nurses was rated at 3.17 out of a 5-point scale. Comparing this finding was difficult due to the lack of prior research investigating nurses’ gender sensitivity. However, studies that targeted nursing students by Woo and Yoo [29], and Ju and Lee [27] showed slight differences with average scores of 3.19 and 3.28, respectively. It should be noted that the study by Ju and Lee [27] involved only female nursing students, highlighting a gender difference. The relatively high gender sensitivity among nursing students is believed to be due to the inclusion of education on gender sensitivity in the nursing curriculum and their exposure to the latest theoretical knowledge and gender-related issues, more so than nurses in the clinical setting.

In this study, the correlation between childhood domestic violence experiences and the perception of sexism was examined before identifying the factors affecting nurses’ gender sensitivity. As a result, a negative correlation with gender sensitivity was shown by the perception of sexism. This reaffirms the importance of clear organizational management measures to reduce perceptions of sexism and increase gender sensitivity, considering the scores of gender discrimination perception together [30]. Furthermore, hierarchical regression analysis was conducted, and it was found that age and the perception of sexism are significant factors influencing gender sensitivity. The finding that age is a significant factor influencing gender sensitivity reflects generational cultural differences, suggesting that divergent from traditional societal expectations related to gender roles, more open and flexible approaches are affecting gender perceptions and attitudes across different age groups. Accordingly, the higher level of gender sensitivity among the younger age groups could lead to generational conflict, indicating the need for education on gender sensitivity to be received by all nurses. Additionally, the content of such education should vary according to rank to address these differences effectively.

The perception of sexism was confirmed as a factor influencing gender sensitivity. The perception of sexism is largely attributed to unconscious bias, impacting human interactions and leading to serious forms of discrimination [29]. Particularly, the perception of sexism among nurses can affect interactions between nurses, the provision of medical services, and clinical decision-making, which can hinder the adoption of fair and equal nursing attitudes [31]. Therefore, education that enables nurses to recognize discriminatory situations through their own experiences and observations and to develop the ability to think critically about them is necessary [8]. Additionally, as identified in the study by Stamarski and Son Hing [32], since the perception of sexism is influenced by society and culture, the building of capacity for improving discriminatory perceptions at the organizational level and commitment to changing organizational culture are necessary. This implies that within nursing organizations, the emphasis on cultural awareness and capacity development of leaders is seen as crucial components [33]. The gender sensitivity of nursing leaders is essential for providing tailored care that meets the needs of patients of diverse genders within the organization, and for creating an inclusive and non-discriminatory organizational culture that enables all nurses to grow equally.

In this study, based on the theoretical foundation that concepts of gender roles and stereotypes are established during childhood, the extent of childhood experience of domestic violence among nurses was examined as a variable for gender sensitivity. However, the results were not found to be significant. The extent of childhood experience of domestic violence is a difficult subject for individuals to express and is thought to vary according to social and cultural backgrounds, with different standards being applied to the experiences of domestic violence. Therefore, the establishment of a universal consensus on the concept of childhood experience of domestic violence is needed first. Additionally, the perception of sexism measurement tool used in this study has previously established validity and reliability through Lee’s [34] research on generational perceptions of sexism among Korean men and women. However, this tool is not fully adapted to Korean culture, suggesting the need for further research to overcome these cultural adaptation limitations and more extensively validate the tool’s universality.

The gender sensitivity of nurses is recognized as an important concept for changes in professional organizational culture [35]. Gender sensitivity among nurses is recognized as an important concept for changing professional organizational culture. However, limited prior research on gender sensitivity in nurses makes it difficult to fully understand it. Therefore, further research on nurses’ gender sensitivity is necessary. Additionally, gender sensitivity should be an essential part of the education on gender equality awareness and attitudes for nurses, and a structured educational system that allows for continuous exposure should be designed. In this context, this study is expected to explore concrete measures to enhance gender sensitivity in nursing practice and contribute to the improvement of discriminatory perceptions and the change of organizational culture within nursing organizations.

## Conclusions

Age and perception of sexism were identified as factors affecting nurses’ gender sensitivity, accounting for 42.7% of the explanatory power. Therefore, to enhance nurses’ gender sensitivity, the acknowledgment of generational cultural differences within nursing organizations and the adoption of a flexible and open approach are necessary. Furthermore, the development of cultural awareness and capacity for gender sensitivity among leaders within nursing organizations should be prioritized to address perceptions of sexism. Through this, an environment that embraces gender diversity and is free from discrimination can be created by leaders within nursing organizations, thereby laying the foundation for delivering high-quality nursing care in the field. Based on this study, the development and validation of the effectiveness of gender sensitivity training programs through follow-up research are recommended.

## Data Availability

The datasets used and analyzed during the current study available from the corresponding author on seasonable request.
